# Evaluation of FGFR inhibitor ASP5878 as a drug candidate for achondroplasia

**DOI:** 10.1038/s41598-020-77345-y

**Published:** 2020-12-01

**Authors:** Tomonori Ozaki, Tatsuya Kawamoto, Yuki Iimori, Nobuaki Takeshita, Yukiko Yamagishi, Hiroaki Nakamura, Masazumi Kamohara, Kaori Fujita, Masayuki Tanahashi, Noriyuki Tsumaki

**Affiliations:** 1 Cell Induction and Regulation Field, Department of Clinical Application, Center for iPS Cell Research and Application, Kyoto University, 53 Kawahara-cho, Shogoin, Sakyo-ku, Kyoto, 606-8507 Japan; 2 Department of Orthopaedic Surgery, Osaka City University Graduate School of Medicine, Osaka, Japan; 3 Drug Discovery Research, Astellas Pharma Inc., Ibaraki, Japan

**Keywords:** Drug discovery, Drug safety, Pluripotent stem cells, Cell signalling, Rheumatic diseases, Musculoskeletal abnormalities, Diseases, Growth disorders, Medical research, Drug development

## Abstract

Achondroplasia is caused by gain-of-function mutations in *FGFR3* gene and leads to short-limb dwarfism. A stabilized analogue of C-type natriuretic peptide (CNP) is known to elongate bone by interacting with FGFR3 signals and thus is a promising drug candidate. However, it needs daily administration by percutaneous injection. FGFR inhibitor compounds are other drug candidates for achondroplasia because they directly fix the mutant protein malfunction. Although FGFR inhibitors elongate the bone of model mice, their adverse effects are not well studied. In this study, we found that a new FGFR inhibitor, ASP5878, which was originally developed as an anti-cancer drug, elongated the bone of achondroplasia model male mice at the dose of 300 μg/kg, which confers an AUC of 275 ng·h/ml in juvenile mice. Although ASP5878 was less effective in bone elongation than a CNP analogue, it is advantageous in that ASP5878 can be administered orally. The AUC at which minimal adverse effects were observed (very slight atrophy of the corneal epithelium) was 459 ng·h/ml in juvenile rats. The positive discrepancy between AUCs that brought efficacy and minimal adverse effect suggests the applicability of ASP5878 to achondroplasia in the clinical setting. We also analyzed effects of ASP5878 in a patient-specific induced pluripotent stem cell (iPSC) model for achondroplasia and found the effects on patient chondrocyte equivalents. Nevertheless, cautious consideration is needed when referring to safety data obtained from its application to adult patients with cancer in clinical tests.

## Introduction

Achondroplasia (ACH) is the most frequent chondrodysplasia, and its condition leads to disproportionate short-limb dwarfism, occurring with a frequency of 1 in 15–25,000^1,2^. Proximal segments in the limbs are most affected, a phenotype called rhizomelia. In addition, ACH can result in foramen magnum stenosis and spinal canal stenosis, which often cause neurological symptoms that lead to debilitating conditions.

Mutations in the gene encoding fibroblast growth factor receptor 3 (*FGFR3*) were identified in patients with ACH^3,4^. FGFR3 functions as a transmembrane receptor tyrosine kinase, and mice deficient for FGFR3 show skeletal overgrowth^5^. This mouse phenotype suggests that FGFR3 is a negative regulator of endochondral bone formation in growth plate cartilage and thus that the mutations causing ACH are gain-of-function mutations. *FGFR3* mutations have also been found in other chondrodysplasia conditions including thanatophoric dysplasia^6^, and all related conditions are collectively called FGFR3 chondrodysplasias^7^.

Therapeutic strategies aimed at decreasing excessive FGFR3 signals have been investigated ^8^. The application of c-type natriuretic peptide (CNP)^9^, stabilized forms of CNP^10,11^, parathyroid hormone (PTH)^12^, FGFR3-binding peptides^13^, soluble FGFR3^14^, meclizine^15^, statin^16^ and FGFR inhibitors^17^ could all decrease FGFR signals in cell models and elongated bone length in mouse models of FGFR3 chondrodysplasia.

The most promising therapy so far for the treatment of ACH is the use of a stabilized form of CNP called vosoritide (BMN-111). CNP attenuates the mitogen-activated protein kinase (MAPK) signals activated by FGFR3. A dose-dependent increase in the annualized growth velocity was observed with vosoritide in a phase 2 clinical trial^18^, and vosoritide is currently in a phase 3 clinical trial (ClinicalTrials.gov NCT02055157). However, vosoritide needs to be administered daily through subcutaneous injections that burden pediatric and juvenile patients considering that treatment would last for years.

The most rational therapeutic strategy is to target FGFR3 using an FGFR tyrosine kinase inhibitor (TKI). Because the same genetic lesions leading to FGFR3-related skeletal disorders cause FGFR3-driven cancers (e.g., bladder tumors and multiple myeloma), FGFR inhibitors that have been investigated for FGFR3-driven cancers can be repurposed for the treatment of ACH. Although inhibitors that would specifically inhibit FGFR3 but not other FGFRs (FGFR1, FGFR2 and FGFR4) are optimal, current FGFR inhibitors are pan-specific (inhibit all FGFRs). NVP-BGJ398 is an FGFR inhibitor that was shown to rescue the skeletal phenotype of ACH model mice^17^. Conducted under the same protocol with the same mouse model, NVP-BGJ398 (2 mg/kg body weight) elongated bone more than vosoritide (800 μg/kg body weight)^17^. However, information on the pharmacokinetics and toxicity of NVP-BGJ398, which are necessary to evaluate the margin of safety, are limited to adult animals and not available for juvenile animals^19^.

ASP5878 is a novel pan-specific FGFR inhibitor under development^20^. The oral administration of ASP5878 induces tumor regression in xenograft mouse models for hepatocellular carcinoma^20^ and urothelial cancer^21^, both of which harbor *FGFR3* gene alternations. A phase I study enrolling adult patients with urothelial carcinoma, hepatocellular carcinoma, or squamous cell lung carcinoma with FGFR genetic alterations has been conducted in order to identify the dose-limiting toxicity (DLT), maximum tolerated dose, and recommended dose of ASP5878^22^. In the present study, we aimed at evaluating the applicability of ASP5878 for the treatment of ACH. For this purpose, we administered ACH model mice with various amounts of ASP5878 in order to determine the minimum amount of ASP5878 that rescues the dwarf phenotype. We next performed a pharmacokinetic study in juvenile mice and rats and a toxicity test in juvenile rats in order to determine the margin between the dose that rescues the skeletal phenotype and the dose that causes adverse events. We finally added ASP5878 to a patient-specific induced pluripotent stem cell (iPSC) model for ACH^16^ in order to confirm the effects on patient chondrocyte equivalents.

## Results and discussion

### Pharmacokinetic study in juvenile mice

**Figure 1:**
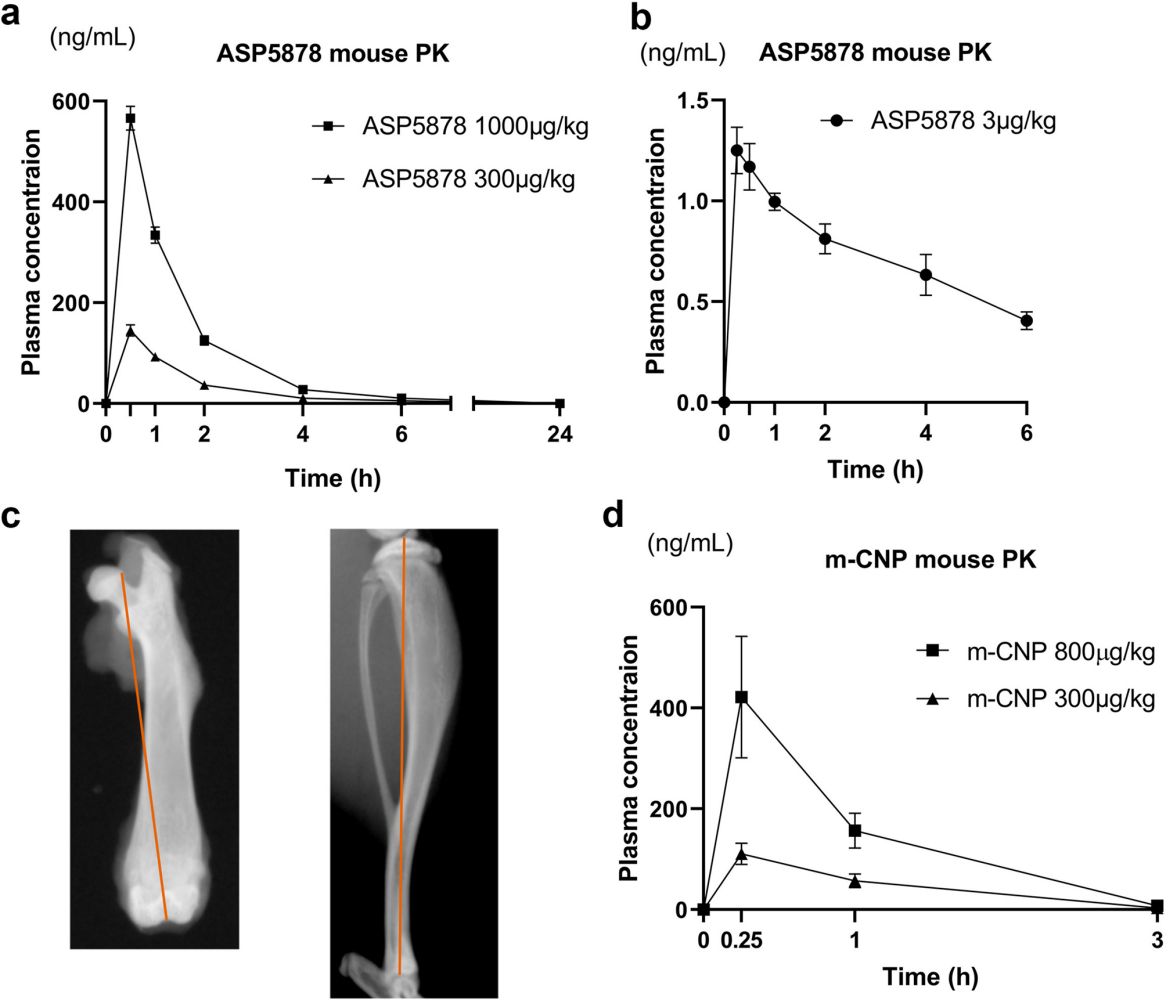
Pharmacokinetic study of ASP5878 and m-CNP in FVB/NJcl mice. **(a)** Plasma concentrations of ASP5878 after the subcutaneous administration of 300 and 1000 μg/kg ASP5878 into 23-day-old normal male mice. **(b)** Plasma concentrations of ASP5878 after the subcutaneous administration of 3 μg/kg ASP5878 into 22-day-old normal male and female mice. **(c)** Measurements of the lengths of the femur and tibia. Red lines indicate the distances measured to designate the lengths of the femur and tibia. **(d)** Plasma concentrations of m-CNP after the subcutaneous administration of 300 and 800 μg/kg m-CNP into 7-week-old normal male mice. Error bars denote the means ± s.d. (n = 3).

Patients with ACH should be treated during stages in which the bone grows (infant, pediatric and juvenile), but pharmacokinetic data for ASP5878 is limited to adults^20^. Thus, we first analyzed the plasma concentration of ASP5878 in 23-day-old FVB/NJcl mice, which corresponds to 6.3-year-old humans^23^. The areas under the plasma concentration–time curve over 24 h (AUC(24)) were 275 and 885 ng·h/ml for 23-day-old male mice that received subcutaneous injection of ASP5878 at dosages of 300 and 1000 μg/kg body weight, respectively (Fig. 1a). The AUC(6) for the administration of 3 μg/kg ASP5878 was 4.39 ng·h/ml (Fig. 1b).

### Effect of ASP5878 administration on bone growth in 21- to 43-day-old ACH model mice

*Fgfr3*^*Ach*^ mice are model mice expressing murine *Fgfr3* cDNA containing the mutation which corresponds to the G380R mutation found in ACH in chondrocytes^24^. We prepared a large number of *Fgfr3*^*Ach*^ mice by in vitro fertilization one time for each experiment. In all three experiments, after separating the mice by sex and genotype, we weighted and divided the *Fgfr3*^*Ach*^ mice into groups so as the differences in averages and standard deviations in weight between groups was minimal at the start of the drug administration. For the wild-type littermate group, we selected mice whose weights were nearest to the median weight of 21-day-old wild-type mice. We subcutaneously injected each group of mice with various amounts of modified CNP peptide (m-CNP) or ASP5878 five times a week from 21- to 42-days-old, sacrificed them at 43-days-old, took X ray images to measure bone length (Fig. 1c), and dissected the knee joints for histological analysis. Twenty-one- to 43-day-old mice correspond to 5.8- to 11.8-year-old humans^23^. The background strain of mice used in these experiments was an F1 hybrid between FVB and C57BL/6.

**Figure 2:**
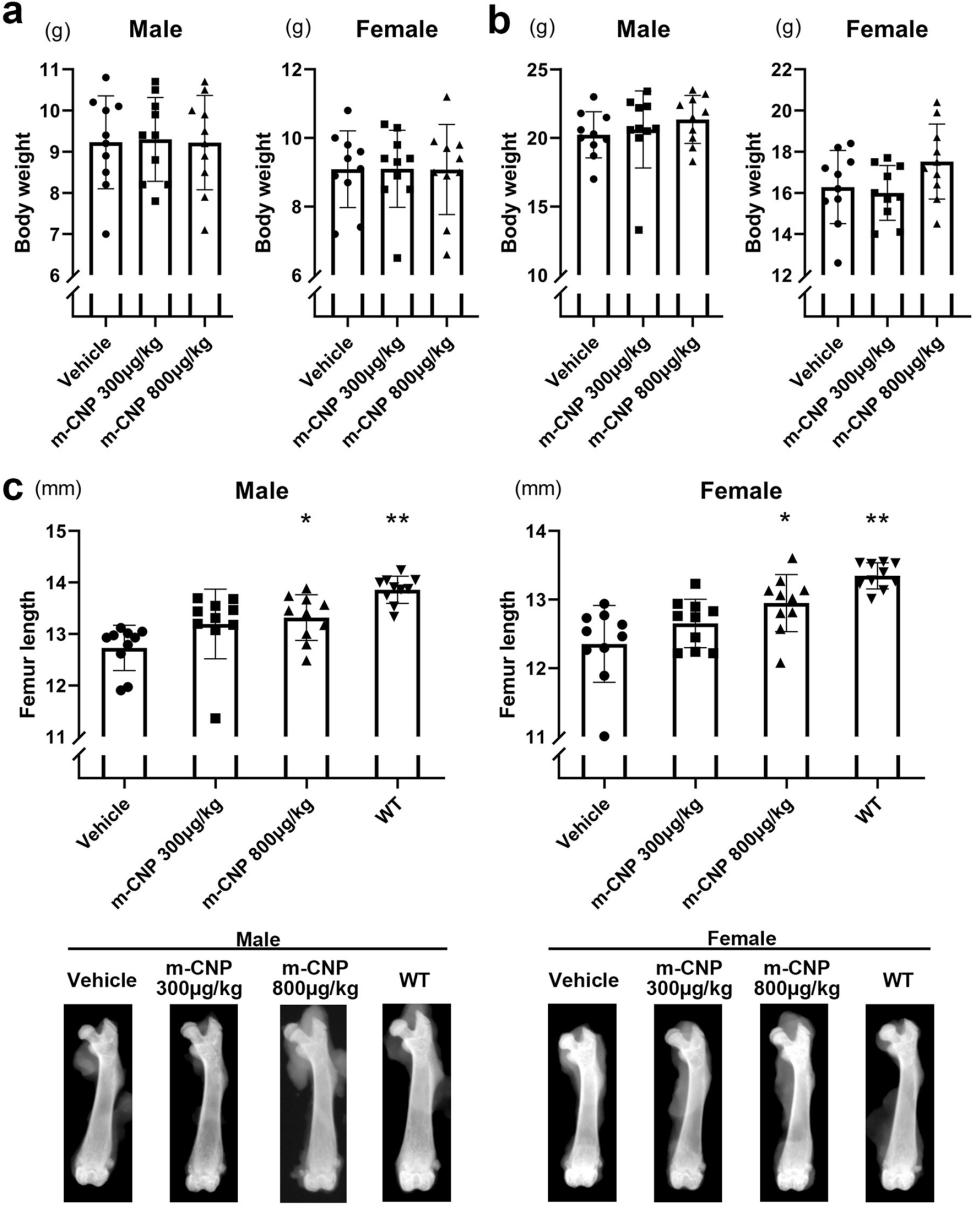
Effects of 300 and 800 μg/kg m-CNP administration for 3 weeks on femur length in *Fgfr3*^*Ach*^ mice. **(a)** Body weight just before the administration to 21-day-olds. **(b)** Body weight after the administration to 43-day-olds. **(c)** Top, femur length after the administration to 43-day-olds. For the group of wild-type littermates, we selected 10 mice whose weights were nearest to the median of the 21-day-old transgenic mice. The wild-type mice were treated with vehicle, sacrificed at 43-days-old, and subjected to bone measurements. Bottom, the images of the femur with median length in each group are shown. Error bars denote the means ± s.d. *P < 0.05; **P < 0.01 compared to vehicle by one-way ANOVA with post-hoc Tukey HSD test.

The first experiment was conducted to validate our procedure by using m-CNP, which was chemically synthesized and the same amino acid sequence as vosoritide. We confirmed that the plasma concentration of m-CNP after subcutaneous administration into wild-type mice (Fig. 1d) was comparable to the plasma concentration of vosoritide after subcutaneous administration reported previously^11^. Thirty male and 30 female 21-day-old *Fgfr3*^*Ach*^ mice were divided into three groups, between which the weights were comparable (Fig. 2a). Based on the group, we administered the mice 0, 300 or 800 μg/kg m-CNP by subcutaneous injection until 42-days-old and sacrificed them at 43-days-old, when there was no significant difference in weight (Fig. 2b). Male and female *Fgfr3*^*Ach*^ mice injected with 800 μg/kg m-CNP had longer femurs than the respective *Fgfr3*^*Ach*^ mice injected with vehicle (Fig. 2c). Injection of 300 μg/kg m-CNP substantially elongated the male and female femurs, although the differences were not significant with the sample numbers we employed. These results suggested that our experimental system using the ACH model mice was feasible.

**Figure 3:**
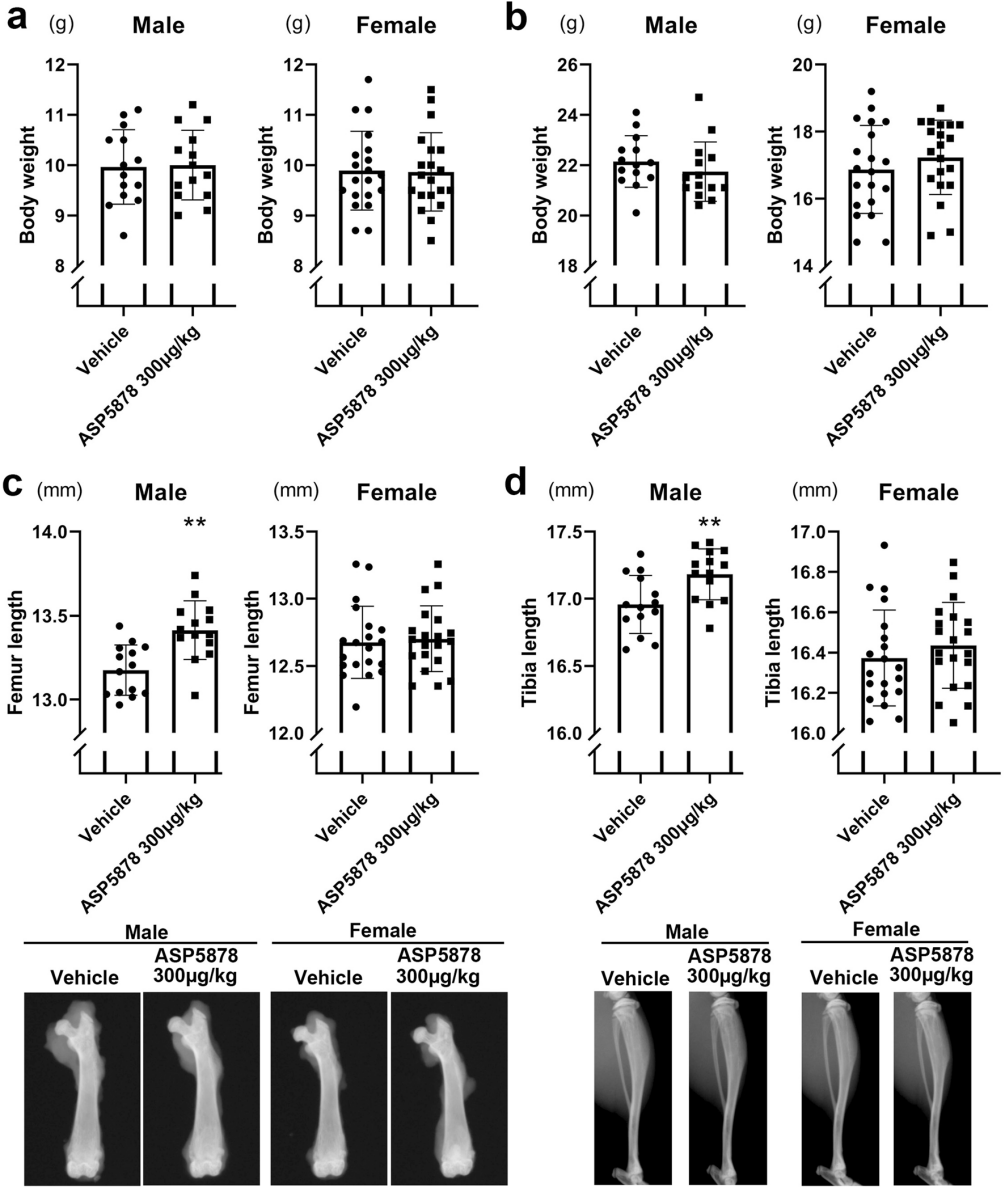
Effects of 300 μg/kg ASP5878 administration for 3 weeks on bone length in *Fgfr3*^*Ach*^ mice. **(a)** Body weight just before the administration to 21-day-olds. **(b)** Body weight after the administration to 43-day-olds. **(c)** Top, femur length after the administration to 43-day-olds. Bottom, the images of the femur with median length in each group are shown. **(d)** Top, tibia length after the administration to 43-day-olds. *Bottom*, the images of the tibia with median length in each group are shown. Error bars denote the means ± s.d. *P < 0.05; **P < 0.01 compared to vehicle by the t-test.

In the second experiment, we treated the *Fgfr3*^*Ach*^ mice with 300 μg/kg ASP5878 or vehicle. Twenty-eight male and 40 female *Fgfr3*^*Ach*^ mice were respectively divided into two groups of comparable weights at 21-days-old (Fig. 3a) and then treated with 300 μg/kg ASP5878 or vehicle. At 43-days-old, there was no significant difference in weight between groups within gender (Fig. 3b). The lengths of the femurs and tibiae in male *Fgfr3*^*Ach*^ mice with 300 μg/kg ASP5878 were significantly longer than those with vehicle, but there was no significant difference in the lengths of these bones in the two female groups (Fig. 3c,d).

Regarding synchondrosis, we measured the length of the cranial base in micro-CT images. The administration of 300 μg/kg ASP5878 significantly elongated the length of the cranial base more than the vehicle in female mice but not in male mice (see Supplementary Fig. S1a online). To explore the reason, we performed micro-CT analysis and found that the synchondrosis was closed in 4 out of 8 male 21-year-old *Fgfr3*^*Ach*^ mice, which was when we started the administration of ASP5878. Synchondrosis was only slightly open in all 8 female *Fgfr3*^*Ach*^ mice. However, synchondrosis was clearly open in the wild-type mice (see Supplementary Fig. S1b online).

**Figure 4:**
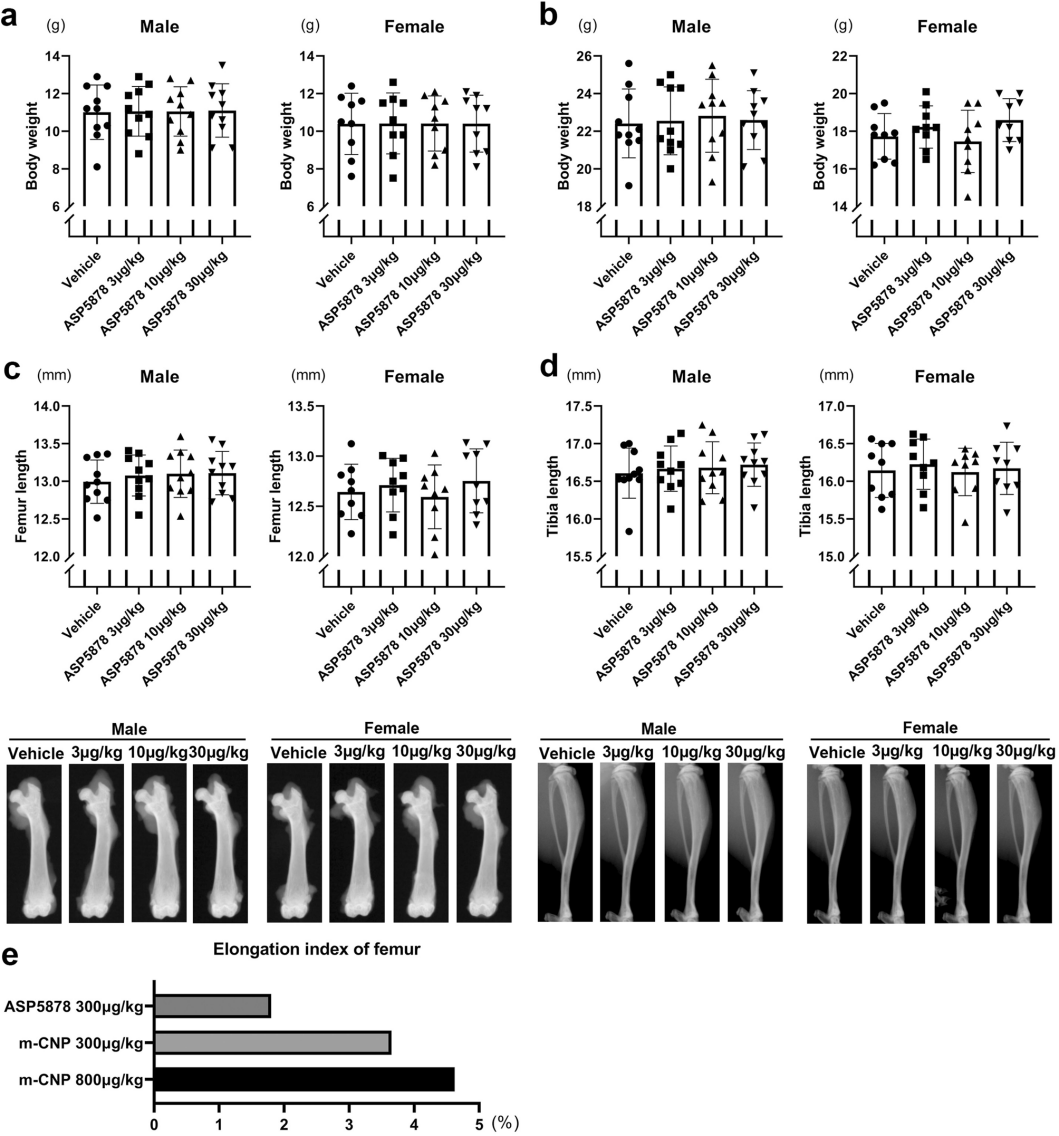
Effects of 3, 10 and 30 μg/kg ASP5878 administration for 3 weeks on bone length in *Fgfr3*^*Ach*^ mice. **(a)** Body weight just before the administration to 21-day-olds. **(b)** Body weight after the administration to 43-day-olds. **(c)** Top, femur length after the administration to 43-day-olds. Bottom, the images of the femur with median length in each group are shown. **(d) **Top, tibia length after the administration to 43-day-olds. Bottom, the images of the tibia with median length in each group are shown. **(e)** Comparison of 300 μg/kg ASP5878 administration and m-CNP administration on femur length in male *Fgfr3*^*Ach*^ mice. We calculated the elongation length by subtracting the mean bone lengths of mice treated with vehicle from those of mice treated with ASP5878 or m-CNP. We further divided the elongation length by the mean bone length of the mice treated with vehicle and designated the quotient as the elongation index. Error bars denote the means ± s.d. *P < 0.05; **P < 0.01 compared to vehicle by one-way ANOVA with post-hoc Tukey HSD test.

The third experiment was conducted to determine the minimum dose of ASP5878 that elongates bone in *Fgfr3*^*Ach*^ mice. Forty male and 36 female *Fgfr3*^*Ach*^ mice were respectively divided into four groups of comparable weights at 21-days-old (Fig. 4a). There were no significant differences in weight at 43-days-old (Fig. 4b). The lengths of the femurs or tibiae of *Fgfr3*^*Ach*^ mice treated with 3, 10 or 30 μg/kg ASP5878 were not significantly different from those treated with vehicle within each gender (Fig. 4c,d). The results from the second and third experiments suggest that the minimum effective dose of ASP5878 resides somewhere between 30 and 300 μg/kg. ASP5878 was less effective at bone elongation than m-CNP (Fig. 4e).

Finally, no mice died prior to the end of the experiments.

### Effects of ASP5878 on the thickness of growth plate cartilage

**Figure 5:**
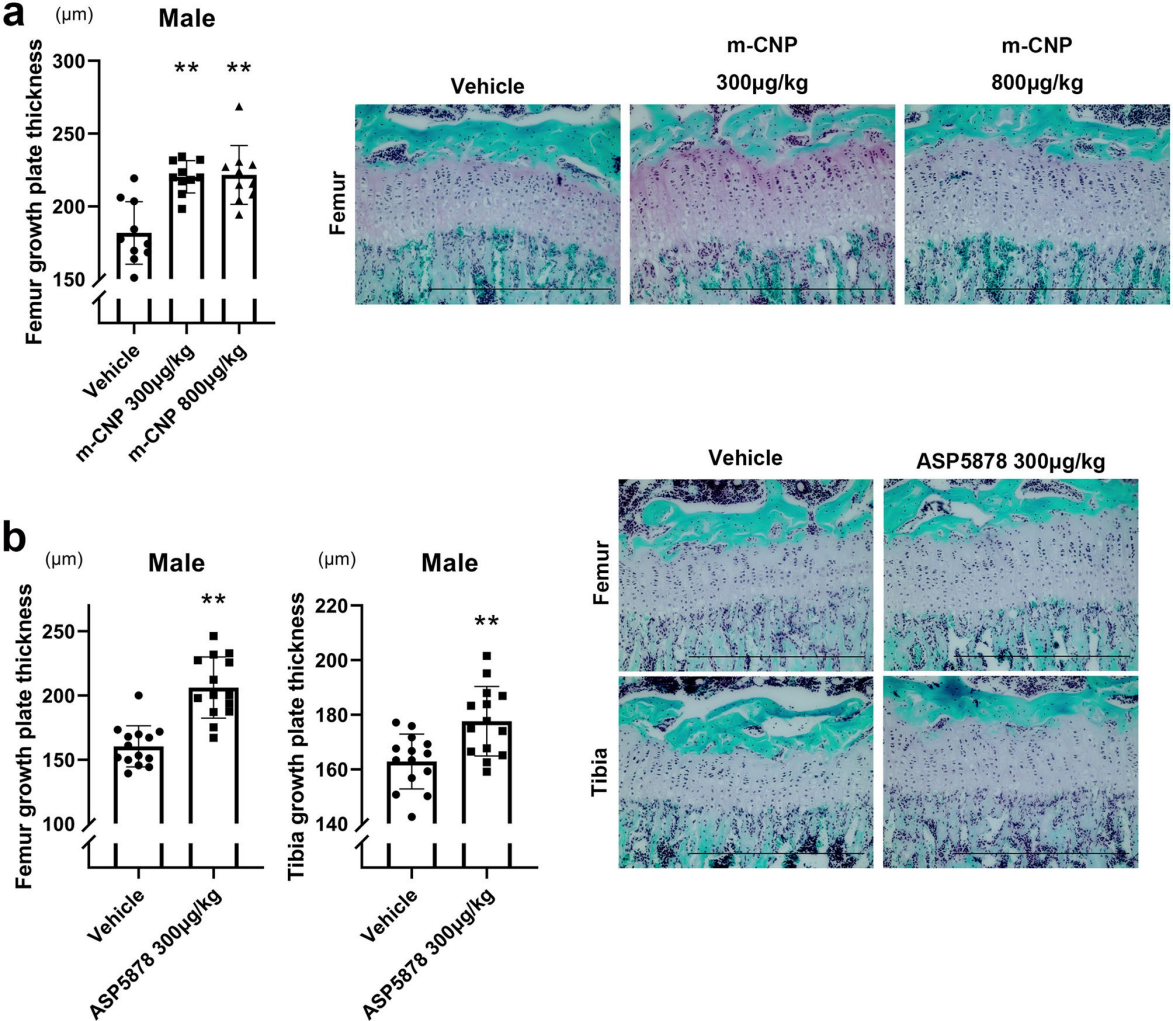
Thickness of growth plate cartilage in *Fgfr3*^*Ach*^ mice treated with m-CNP or ASP5878. Mice at 21-days-old were treated for 3 weeks. **(a)** Left, thickness of growth plate cartilage in male *Fgfr3*^*Ach*^ mice treated with m-CNP. Right, histology of the growth plates. **(b)** Left, thickness of growth plate cartilage in male *Fgfr3*^*Ach*^ mice treated with ASP5878. Right, histology of the growth plates. Error bars denote the means ± s.d. *P < 0.05; **P < 0.01 compared to vehicle by one-way ANOVA with post-hoc Tukey HSD Test. Scale bars, 500 μm.

After measuring the bone length, growth plate cartilage was histologically analyzed. The administration of 300 or 800 μg/kg m-CNP increased the thickness of the growth plate cartilage in the distal femur in male *Fgfr3*^*Ach*^ mice (Fig. 5a). The administration of 300 μg/kg ASP5878 increased the thickness of the growth plate cartilage in the distal femur and in the proximal tibia in male *Fgfr3*^*Ach*^ mice (Fig. 5b).

Immunohistochemical analysis indicated that the expression levels of phosphorylated p44/42 MAPK (Erk1/2), a downstream target of FGFR3 signaling, was decreased in growth plate chondrocytes in the proximal tibia of *Fgfr3*^*Ach*^ mice treated with 300 μg/kg ASP5878 compared with those treated with the vehicle (see Supplementary Fig. S2a online). There was no obvious difference in the expression of total p44/42 MAPK between mice treated with 300 μg/kg ASP5878 and vehicle.

### Toxicity test for ASP5878 in juvenile rats

A toxicity study in which 3-week-old rats were administered orally and daily for two weeks 30 μg/kg body weight ASP5878 found no adverse effects. The administration of 300 μg/kg body weight ASP5878 caused only very slight atrophy of the corneal epithelium in two of six animals for each sex. No test article-related deaths or moribundities occurred. In addition, no test article-related changes were noted in clinical signs, body weight, food consumption, hematology, blood chemistry, gross pathology, or organ weights in any group (see Supplementary Table S1). Although it is very slight, corneal changes need attention. However, a human clinical trial in which 40 mg ASP5878 was administered twice daily found no adverse event observed in the cornea^22^.

**Figure 6:**
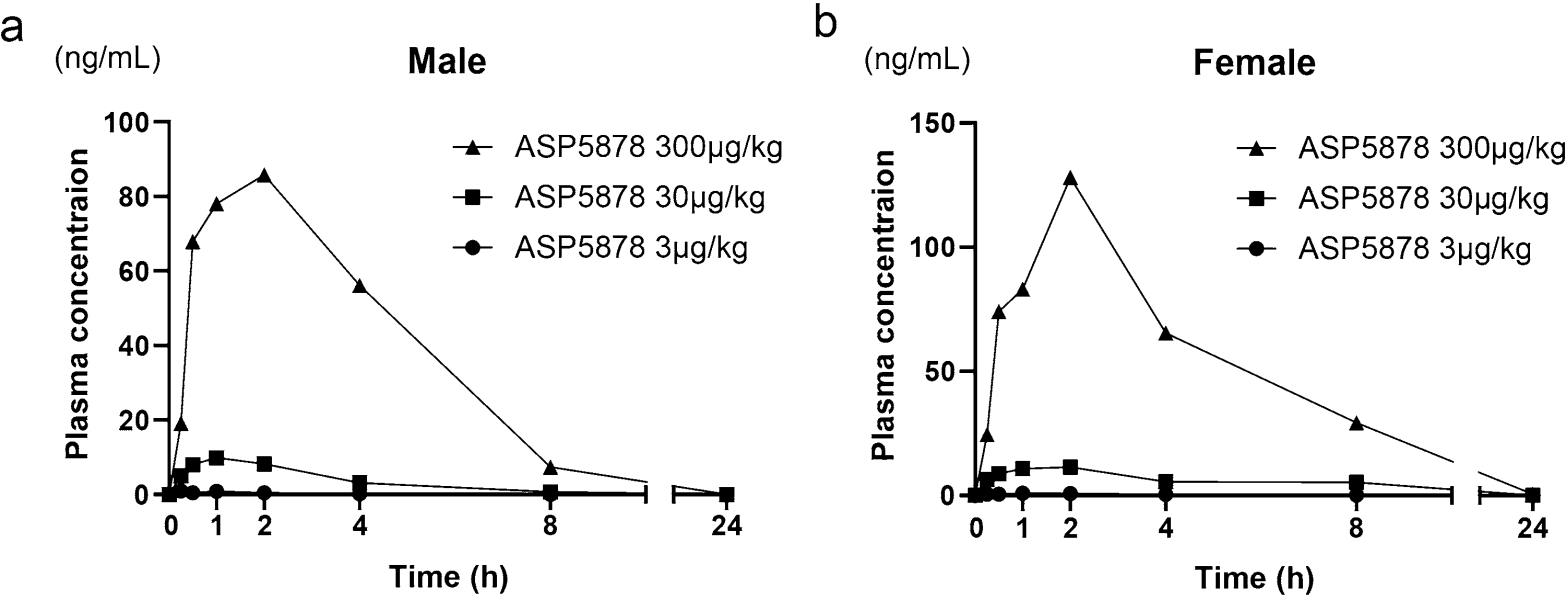
Plasma concentrations of ASP5878 in satellite rats orally administered for 2 weeks. **(a) **Plasma concentration of ASP5878 after the last dosing of 3, 30 and 300 μg/kg ASP5878 into 21-day-old normal male rats. **(b) **Plasma concentration of ASP5878 after the last dosing of 3, 30 and 300 μg/kg ASP5878 into 21-day-old normal female rats.

Plasma mean AUC(24) levels of 300 μg/kg ASP5878 on the last day in satellite male and female rats were 459 and 782 ng·h/ml, respectively (Fig. 6).

### Effects of ASP5878 in a patient-specific iPSC disease model for ACH

A patient-specific iPSC disease model for ACH was previously described^16^. According to that previous study, the chondrogenic differentiation of iPSCs from healthy individuals induces chondrocytes. Further culture in three-dimensional suspension leads the chondrocytes to produce extracellular matrix (ECM), resulting in cartilaginous particles in which chondrocytes are embedded in cartilage ECM. On the other hand, the chondrogenic differentiation of iPSCs from patients with ACH (ACH-iPSCs) results in the formation of particles that are not cartilaginous.

**Figure 7:**
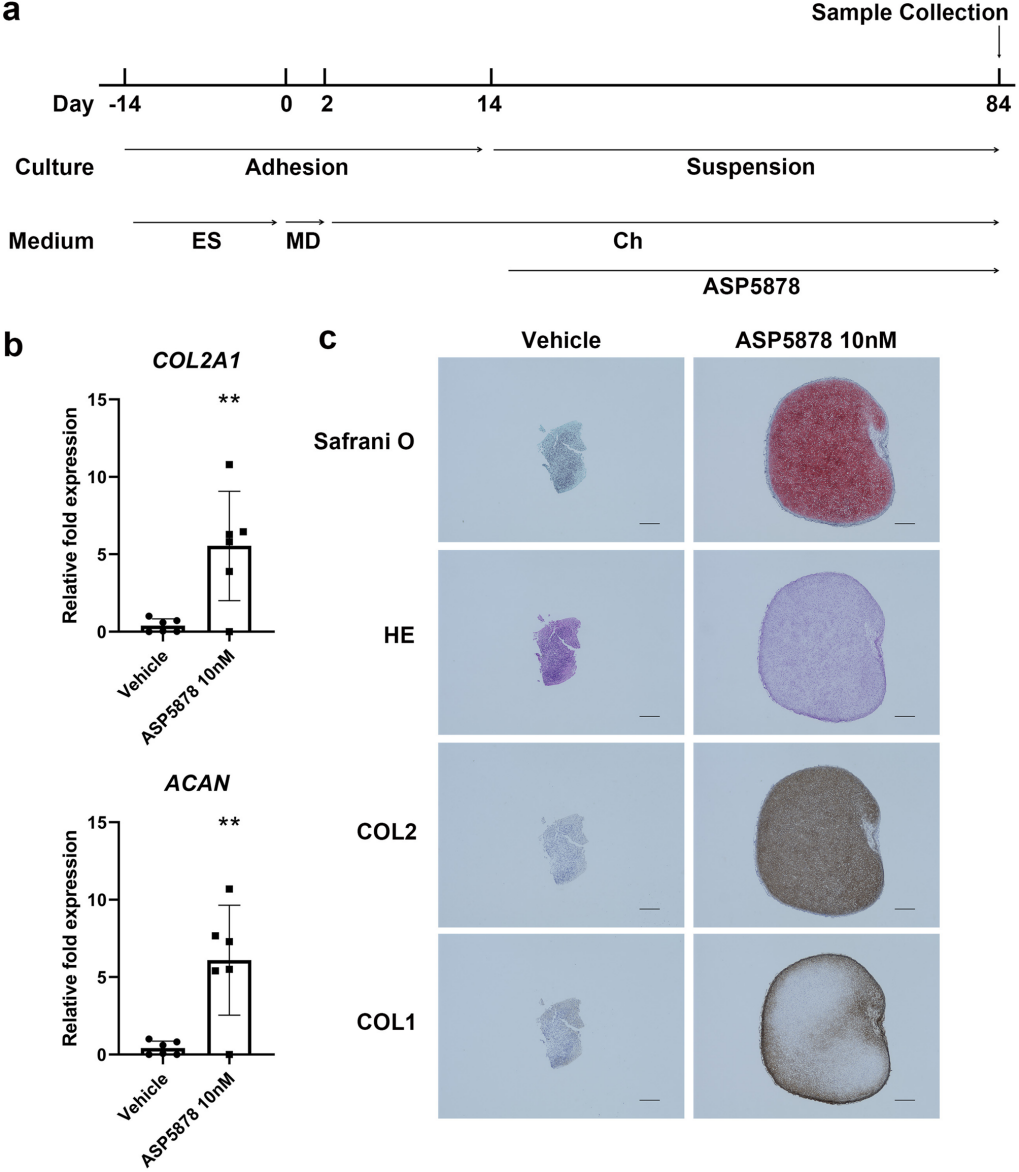
Effects of ASP5878 in a patient-specific iPSC disease model for ACH. **(a)** Scheme showing the chondrogenic differentiation of ACH-iPSCs. After chondrogenic differentiation, the cells were transferred to suspension culture. ASP5878 or vehicle was added from day 15. Particles were subjected to analysis on day 84. *ES*, embryonic stem cell medium; *MD* mesodermal medium, *Ch* chondrogenic medium. **(b)** Results of a real-time RT-PCR expression analysis of marker genes. Three out of four independent experiments showed similar results. Error bars denote the means ± s.d. *P < 0.05; **P < 0.01 by the t-test. **(c)** Histology of the particles. Scale bars, 300 μm.

The addition of 10 nM ASP5878 during the chondrogenic differentiation of ACH-iPSCs (Fig. 7a) increased the expression levels of the chondrocytic markers *ACAN* and *COL2A1* in the particles (Fig. 7b). Histological analysis confirmed these particles had cartilaginous properties. The ECM was positively stained with Safranin O, which stains glycosaminoglycan, and immunostained with anti-COL2 antibody in the particles formed with ASP5878 but not without (Fig. 7c). The cartilaginous particles were surrounded by perichondrium that expressed COL1, as reported previously^25^.

Additional histological analysis of ACH-iPSC-derived particles revealed that treatment with 10 nM ASP5878 decreased the number of TUNEL-positive cells (see Supplementary Fig. S2b). Immunoreactivity against anti-Ki67 antibody was seldom recognized in the cartilaginous particles cultured in the presence of 10 nM ASP5878, being consistent with the report that chondrocytes in iPSC-derived cartilaginous particles do not proliferate after obtaining cartilaginous properties^25,26^. On the other hand, some nuclei in particles cultured with vehicle are Ki67-positive (see Supplementary Fig. S2b, red arrowheads), suggesting that fibroblastic cells in the particles proliferated in response to excess FGF signaling.

### Evaluation of ASP5878 as a drug candidate for ACH

Our results indicated that ASP5878 was less effective at bone elongation than m-CNP, which has the same amino acid sequence as vosoritide. However, in terms of the route of administration, vosoritide needs subcutaneous injection, whereas ASP5878 can be taken orally, which reduces the burden on pediatrics patients.

We initially tried oral gavage using an intubation tube to administer the mice but failed, because the *Fgfr3*^*Ach*^ mice were small and their spine was curved severely at 21-days-old. Therefore, we employed subcutaneous injection for the administration. We consider the amount of ASP5878 that reached growth plate cartilage depends on the concentration of ASP5878 in the blood regardless of the administration route. Therefore, we measured the concentrations of ASP5878 in the blood and compared the effects.

The minimum dose of ASP5878 through subcutaneous injection that elongated bones in male 21- to 43-day-old *Fgfr3*^*Ach*^ mice was 300 μg/kg. The estimated AUC(24) in 23-day-old mice was 275 ng·h/ml. On the other hand, a toxicity test revealed that only a slightly adverse event was found in the cornea of juvenile rats, in which the AUC(24) of ASP5878 was 459 ng·h/ml in males and 782 ng·h/ml in females. These values are not less than the AUC(24) value that exerts a bone-elongating effect in *Fgfr3*^*Ach*^ mice (275 ng·h/ml), suggesting that the toxicity profile around the exposure that exerts a bone-elongating effect in *Fgfr3*^*Ach*^ mice (275 ng·h/ml) is expected to be minimal.

Why ASP5878 did not significantly elongate the femur and tibia in female *Fgfr3*^*Ach*^ mice requires further study. The growth plate cartilage of these bones was not closed in either male or female *Fgfr3*^*Ach*^ mice. Although we did not find a significant difference in the lengths of bone between the ASP5878 group and vehicle group in female *Fgfr3*^*Ach*^ mice, the mean length tended to be longer in the ASP5878 group. We suspect that increasing the number of mice or the administration period (beyond 22 days) would lead to a significant difference.

In our ACH-iPSC disease model, ASP5878 was effective at 10 nM, which corresponds to approximately 4 ng/ml in the culture medium. In blood, approximately 80% of ASP5878 is bound to albumin for mouse plasma (in-house data) and sequestered, preventing its action. The C_max_ in 23-day-old mice treated with ASP5878 at the dose that elongated bones in *Fgfr3*^*Ach*^ mice was 145 ng/ml, which corresponds to approximately 30 ng/ml as unbound concentration. Therefore, the range of concentrations of ASP5878 in the mouse experiments in this study covered the ASP5878 concentration that can affect human diseased chondrocytes in vitro.

To conclude, this study showed that bone length can be elongated in juvenile model mice following ASP5878 exposure at doses which were lower than those causing minimal adverse effects in juvenile rats. The assessment of safety is important when moving forward to clinical tests. However, because the administration of ASP5878 to adult patients with cancers showed adverse effects, such as hyperphosphatemia, retinal detachment, diarrhea, and increased alanine aminotransferase, in clinical tests^22^ (information on the clinical trial is also available at https://www.astellas.com/jp/ja/innovation/clinical-trials-results), the application of ASP5878 to juvenile patients with ACH should be cautiously considered.

## Materials and methods

### Ethics statement

All methods were carried out in accordance with relevant guidelines and regulations. Experiments using recombinant DNA was approved by the Recombinant DNA Experiments Safety Committee of Kyoto University. Research involving human subjects was approved by the Ethics Committee of Kyoto University. Written informed consent was obtained from each donor. All animal experiments were approved by the institutional animal committee of Kyoto University.

### Preparation of ASP5878 solution and its pharmacokinetics study in juvenile mice

ASP5878 solution was prepared by dissolving ASP5878 in 5% propyleneglycol and 5% HP-β cyclodextrin (50% (w/v)) in saline at pH 7.0.

In the pharmacokinetic study, the profile of ASP5878 was determined using 22–23-day-old male and female normal FVB/NJcl mice (Japan Clea Inc.) after a single subcutaneous (3, 300 and 1000 μg/kg in mice, n = 3) administration of ASP5878 solution. Plasma was collected at 0.5, 1, 2, 4, 6 and 24 h (for 300 and 1000 μg/kg administration) or 0.25, 0.5, 1, 2, 4 and 6 h (for 3 μg/kg administration) after the administration. The plasma concentration of ASP5878 was measured with LC/MS/MS.

### Preparation of modified CNP solution and its pharmacokinetics study in mice

Modified CNP (m-CNP) was chemically synthesized by Peptide Institute, Inc. (Osaka, Japan). The amino acid sequence, which is the same as vosoritide, is NH2-PGQEHPNARKYKGANKKGLSKGCFGLKLDRIGSMSGLGC-COOH (C23–C39 S–S bond). The peptide was 99.2% pure, and masses of the peptide were confirmed by LC/MS. The m-CNP solution was prepared by dissolving peptide-CNP in 30 mM acetic acid, pH 4.0, containing 10% sucrose and 1% benzyl alcohol.

In the pharmacokinetic study, the profile of peptide-CNP was determined in 7-week-old male normal FVB/NJcl mice (Japan Clea Inc.) after a single subcutaneous (300 and 800 μg/kg in mice, n = 3) administration of m-CNP solution. Plasma was collected by decapitation at 0.25, 1 and 3 h after the administration. The plasma concentration was determined using a competitive ELISA kit (CNP-22, Phoenix Pharmaceutical, Inc.).

### ACH model mice

*Fgfr3*^*Ach*^ transgenic mice were a gift from D.Ornitz (Washington University School of Medicine)^24^. We produced a large number of mice by in vitro fertilization. In this study, spermatozoa were collected from male heterozygous *Fgfr3*^*Ach*^ mice (FVB strain). Oocytes were collected from superovulated wild-type female mice (C57BL/6 mice). The spermatozoa were added to the oocytes. Fertilized oocytes were transferred into pseudopregnant mice (ICR).

After the subcutaneous administration of m-CNP solution or vehicle, or ASP5878 solution or vehicle to 21-day-old *Fgfr3*^*Ach*^ transgenic mice for three weeks, the mice were sacrificed and subjected to X-ray imaging (Faxitron X-ray DX-50). Regarding the femur, hindlimbs were dissected and femur was isolated and subjected to X-ray imaging. The lengths of the femur and tibia were measured with ImageJ software (National Institutes of Health). We measured the distance between the lateral metaphyseal end in the femoral head and the bottom of the concave between the femoral condyles, and designated it as the femoral length (Fig. 1c left). We measured the distance between the top of the tibial articular surface and the center of the distal tibial articular surface, and designated it as the tibial length (Fig. 1c right). Then the knee joints were further dissected and processed for histological analysis.

We calculated the elongation length by subtracting the mean bone lengths of mice treated with vehicle from those treated with ASP5878 or m-CNP. We further divided the elongation length by the mean bone length of mice treated with vehicle and designated the quotient as the elongation index.

For examination of the cranial base, we subjected the skulls to a micro-CT imaging system (inspeXio SMX-100CT, Shimadzu, Kyoto, Japan) at 60 kV, 40 μA and 45 μm/voxel with a metal filter. The length of the cranial base was measured using Image J.

### RNA extraction and quantitative real-time RT-PCR

The samples were frozen and homogenized with a Multibead shocker (Yasui Kikai). Total RNA was extracted by using NucleoSpin RNA Plus XS (Macherey–Nagel). The cDNA was synthesized from 100 ng of total RNA by using the ReverTra Ace (Toyobo). Real-time PCR was conducted by using Taqman probes (*ACAN*, Hs01048720_m1; *COL2A1*, Hs01060355_g1; *GAPDH*, Hs03929097_g1). The RNA expression levels were normalized to the level of *GAPDH*.

### Histological and immunohistochemical analysis

Mouse knee joints were dissected and fixed in 4% paraformaldehyde for 5 days, decalcified in 10% ethylenediaminetetraacetic acid for 21 days, and embedded in paraffin. Three-micrometer sections were prepared and stained with Safranin-O-Fast green-iron hematoxylin.

The particles created from ACH-iPSCs were fixed in 4% paraformaldehyde overnight, embedded in paraffin, and sectioned. Semi-serial sections were stained with Safranin-O-fast green-iron hematoxylin or immunostained with specific antibodies. Primary antibodies were against type I collagen (Southern Biotechnology Associates Inc., code : 1310-01, 1:500), type II collagen (Southern Biotechnology Associates Inc., code: 1320-01, 1:600), anti-phospho-p44/42 MAPK (Erk1/2) antibody (Cell signaling, 4370, 1:100), anti- p44/42 MAPK antibody (Cell signaling, 4695, 1:100), or anti-Ki67 antibody (Abcam, ab16667, 1:100). Immune complexes were detected by using N-Histofine Simple Stain MAX PO (GO) (Nichirei Biosciences, 414351) and DAB (Nichirei Biosciences, 725191) as a chromogen or detected by using secondary antibodies conjugated to Alexa Fluor 488 (Life Technologies, 1:500).

For the TUNEL assay, an in situ cell death detection kit (TMR red; Roche) was used according to the manufacturer’s instructions.

### Exploratory 2 weeks-toxicity study of ASP5878 in juvenile rat

ASP5878 was suspended in 0.5% w/v methylcellulose solution and orally administered once daily for 2 weeks from postnatal day 21 at dose levels of 0 (vehicle control), 3, 30, and 300 μg/kg to six male and six female Crl:CD (SD: Charles River Laboratories Japan Inc.) juvenile rats per group in order to investigate its toxicity. A satellite group (4 males and 4 females in each test article group) was added at each dose level to assess the systemic exposure to ASP5878. The following observations and examinations were performed: clinical signs, body weight, food consumption, hematology, blood chemistry, gross pathology, organ weights, histopathology, and toxicokinetics.

### Chondrogenic differentiation of hiPSCs derived from patients with ACH to create particles

The iPSC line ACH-8858, which was derived from a patient with ACH (ACH-iPSC), was previously reported^16^. Chondrogenically differentiated ACH-iPSCs were further cultured in three-dimensional suspension culture to create cartilaginous particles following a previously described method^16,25^. We added ASP5878 at 10 nM final concentration or vehicle from 15 days after the start of the chondrogenic differentiation. Particles were retrieved on day 84 and subjected to mRNA expression and histological analyses.

### Statistical analysis

Data are shown as averages and standard deviations. Student’s t-test was used for two sample comparisons, and one-way analysis of variance with post-hoc Tukey’s Honest Significant Difference test (one-way ANOVA with post-hoc Tukey HSD Test) was used for multiple comparisons. P-values < 0.05 were considered to be statistically significant. Prism8 (Graphpad) was used for the statistical analysis.

## Supplementary information

Supplementary Information.
