# Supplementary material for: Evaluation of FGFR inhibitor ASP5878 as a drug candidate for achondroplasia
Source: Sci Rep. 2020 Dec 1;10:20915. doi: 10.1038/s41598-020-77345-y (PMC7708468; doi:10.1038/s41598-020-77345-y)
Supplement: Supplementary file 1 — Supplementary Information. [file 41598_2020_77345_MOESM1_ESM.pdf]

Supplementary Information

Evaluation of FGFR inhibitor ASP5878 as a drug candidate for achondroplasia

Tomonori Ozaki, Tatsuya Kawamoto, Yuki Iimori, Nobuaki Takeshita, Yukiko Yamagishi, Hiroaki Nakamura, Masazumi Kamohara, Kaori Fujita, Masayuki Tanahashi, Noriyuki Tsumaki

Supplementary Figure S1

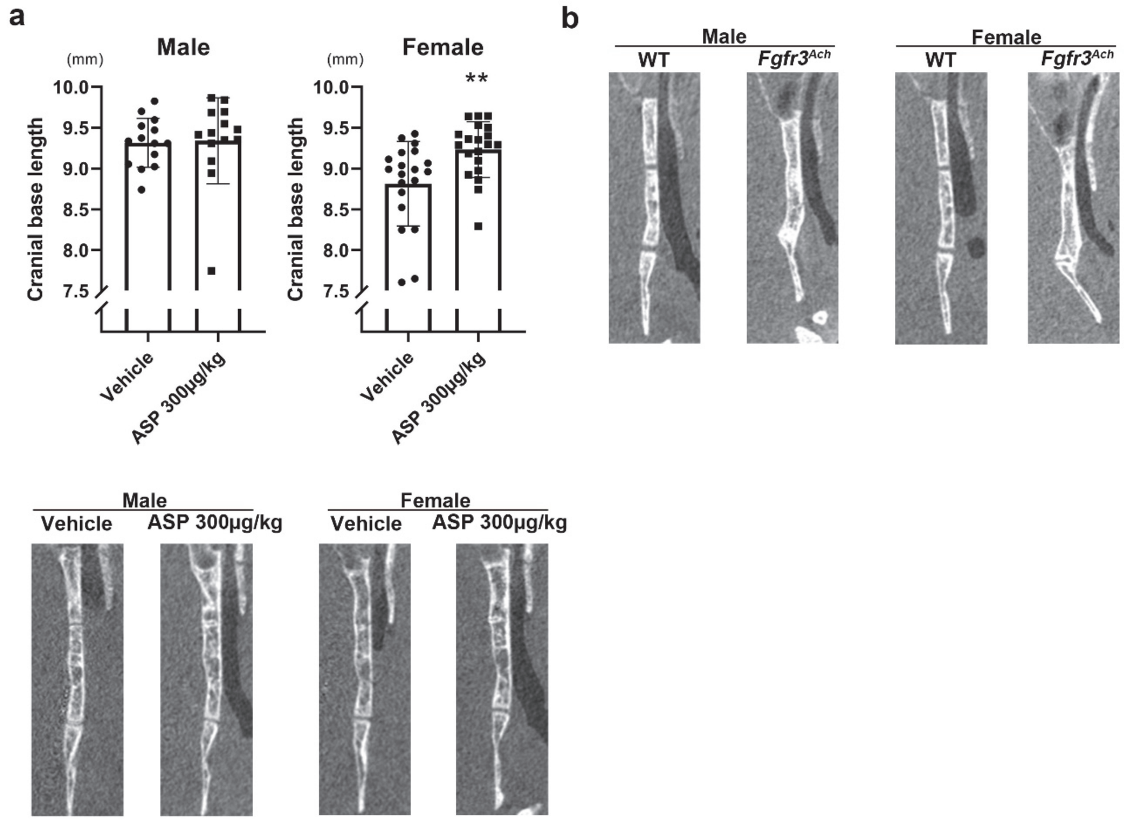

Supplementary Figure S1. Effects of 300 μg/kg ASP5878 administration for 3 weeks on the length of cranial bases in *Fgfr3*^{Ach} mice.

a) *Top*, cranial base length after the administration to 43-day-old *Fgfr3*^{Ach} mice. *Bottom*, the micro-CT images of the cranial base with median length in each group are shown.

b) Micro-CT images of the cranial base of wild-type (WT) and *Fgfr3*^{Ach} mice at 21-days-old.

Error bars denote the means ± s.d. **P < 0.01 compared to vehicle by the t-test.

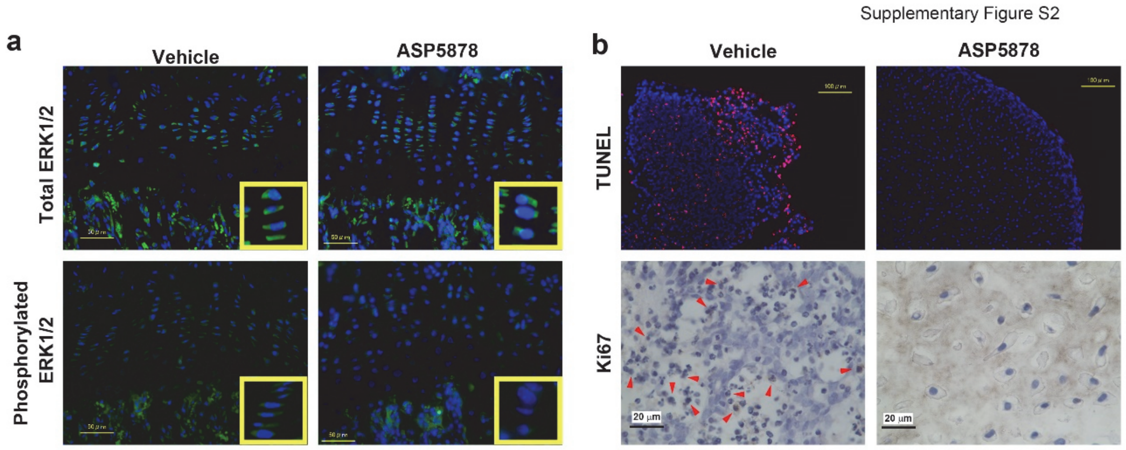

Supplementary Figure S2. Immunohistochemical analysis of the growth plate in *Fgfr3*^{Ach} mice treated with 300 μg/kg ASP5878 and ACH-iPSC-derived particles cultured in the presence of 10 nM ASP5878.

a) Semi-serial sections of the samples in Figure 5a were used. Immunoreactivities against anti-phosphorylated p44/42 MAPK (Erk1/2) antibody (green) and anti-total Erk1/2 antibody (green) were analyzed. The data are representative of three mice.

b) Semi-serial sections of samples in Figure 7c were subjected to TUNEL assay (red) or immunohistochemical analysis for Ki67 (DAB). The data are representative of two ACH-iPSC-derived particles.

Blue color is DAPI.

Supplementary Table S1. Noteworthy Findings in Treatment Period

Daily Dose(μg/kg/day)	0 (Control)		3		30		300	
Number of animals	6/M	6/F	6/M	6/F	6/M	6/F	6/M	6/F
Died or sacrificed moribund	0	0	0	1#	0	0	0	0
Clinical observations	---	---	---	---	---	---	---	---
Body weights	---	---	---	---	---	---	---	---
Food consumption	---	---	---	---	---	---	---	---
Hematology	---	---	---	---	---	---	---	---
Blood chemistry	---	---	---	---	---	---	---	---
Gross pathology	---	---	---	---	---	---	---	---
Organ weights	---	---	---	---	---	---	---	---
Histopathology [No. of animals]<br>Eyeballs<br>Atrophy, corneal epithelium	---	---	---	---	---	---	±[2]	±[2]

F: female; M: male

---: no noteworthy findings; ±: very slight; +: slight; 2+: moderate; 3+: marked

Examination items/organs:

Hematology: Red blood cell count; Hemoglobin; Hematocrit; Reticulocyte ratio; Mean corpuscular volume; Mean corpuscular hemoglobin concentration; Mean corpuscular hemoglobin; White blood cell count; Lymphocytes; Neutrophils; Eosinophils; Basophils; Monocytes; Platelet count; Prothrombin time; Activated partial thromboplastin time

Blood chemistry: Total protein; Albumin; Globulin; Albumin/Globulin ratio; Total bilirubin; Total cholesterol; Triglyceride; Alkaline phosphatase; Aspartate aminotransferase; Alanine aminotransferase; Glucose; Urea nitrogen; Creatinine; Inorganic phosphorus; Calcium; Sodium; Potassium; Chloride

Organ weights: Heart; Spleen; Liver; Kidneys

Histopathology: Heart; Sternum; Sternal bone marrow (control and 300 μg/kg groups); Femur; Femoral bone marrow (control and 300 μg/kg groups); Rib; Thymus; Spleen; Lung; Stomach; Duodenum; Jejunum; Ileum; Liver; Kidney; Thyroid; Adrenal; Testis; Optic nerves; Eyeballs; Femoral Skeletal muscle; Teeth (upper and lower incisor and molar).

#: One female (No. 24) at 3 μg/kg/day was found dead on the morning of Days 2. The cause of death in this animal was considered to be a technical error for dosing, because black focus in the lung and white foamy fluid in the trachea and the bronchi were observed in gross pathology.
